# WNT/β-Catenin Pathway in Soft Tissue Sarcomas: New Therapeutic Opportunities?

**DOI:** 10.3390/cancers13215521

**Published:** 2021-11-03

**Authors:** Esther Martinez-Font, Marina Pérez-Capó, Oliver Vögler, Javier Martín-Broto, Regina Alemany, Antònia Obrador-Hevia

**Affiliations:** 1 Group of Advanced Therapies and Biomarkers in Clinical Oncology, Research Institute of Health Sciences (IUNICS), University of the Balearic Islands, 07122 Palma, Spain; esther.martinez@ssib.es (E.M.-F.); marina.perez@ssib.es (M.P.-C.); oliver.vogler@uib.es (O.V.); 2 Health Research Institute of the Balearic Islands (IdISBa), 07010 Palma, Spain; 3 Medical Oncology Department, Son Espases University Hospital, 07120 Palma, Spain; 4 Group of Clinical and Translational Research, Department of Biology, University of the Balearic Islands, 07122 Palma, Spain; 5 Fundación Jiménez Díaz University Hospital, 28023 Madrid, Spain; jmartin@atbsarc.org; 6 Advanced Therapies & Biomarkers in Sarcoma (ATBSARC), Citius III, Sevilla University, 41013 Sevilla, Spain; 7 Molecular Diagnosis Unit, Son Espases University Hospital, 07120 Palma, Spain

**Keywords:** soft tissue sarcoma, WNT signaling, β-catenin

## Abstract

**Simple Summary:**

The WNT/β-catenin signaling pathway is involved in fundamental processes for the proliferation and differentiation of mesenchymal stem cells. However, little is known about its relevance for mesenchymal neoplasms, such us soft tissue sarcomas (STS). Chemotherapy based on doxorubicin (DXR) still remains the standard first-line treatment for locally advanced unresectable or metastatic STS, although overall survival could not be improved by combination with other chemotherapeutics. In this sense, the development of new therapeutic approaches continues to be an unmatched goal. This review covers the most important molecular alterations of the WNT signaling pathway in STS, broadening the current knowledge about STS as well as identifying novel drug targets. Furthermore, the current therapeutic options and drug candidates to modulate WNT signaling, which are usually classified by their interaction site upstream or downstream of β-catenin, and their presumable clinical impact on STS are discussed.

**Abstract:**

Soft tissue sarcomas (STS) are a very heterogeneous group of rare tumors, comprising more than 50 different histological subtypes that originate from mesenchymal tissue. Despite their heterogeneity, chemotherapy based on doxorubicin (DXR) has been in use for forty years now and remains the standard first-line treatment for locally advanced unresectable or metastatic STS, although overall survival could not be improved by combination with other chemotherapeutics. In this sense, the development of new therapeutic approaches continues to be a largely unmatched goal. The WNT/β-catenin signaling pathway is involved in various fundamental processes for embryogenic development, including the proliferation and differentiation of mesenchymal stem cells. Although the role of this pathway has been widely researched in neoplasms of epithelial origin, little is known about its relevance for mesenchymal neoplasms. This review covers the most important molecular alterations of the WNT signaling pathway in STS. The detection of these alterations and the understanding of their functional consequences for those pathways controlling sarcomagenesis development and progression are crucial to broaden the current knowledge about STS as well as to identify novel drug targets. In this regard, the current therapeutic options and drug candidates to modulate WNT signaling, which are usually classified by their interaction site upstream or downstream of β-catenin, and their presumable clinical impact on STS are also discussed.

## 1. Introduction

Sarcomas are a very heterogeneous group of rare tumors with a very low incidence, accounting for less than 1% of all malignant tumors [1]. These types of tumors originate in the mesenchymal tissue, that is, in the tissue of non-epithelial origin deriving from the embryonic mesoderm. Therefore, they may appear in any part of the body where there is mesenchymal tissue, such as striated or smooth muscle, fibrous tissue or adipose tissue, as well as in cartilage or bones. Historically, these types of tumors are classified, according to the location of the tumor, into two major groups: bone sarcomas (10%) and soft tissue sarcomas (STS) (90%, of which approximately 15% are gastrointestinal stromal tumors (GIST)) [2]. The estimated STS incidence in Europe is five new cases per year per 100,000 inhabitants [3]. According to the latest World Health Organization (WHO) classification from 2020, STS comprise more than 50 different histological subtypes (arranged into 11 major subgroups, as summarized in Table 1), and although they may arise in any part of the body, around 65% are located in the extremities (most commonly in the thigh), 15% in the trunk wall, 5% in the retroperitoneum, and 5% in the head and neck [1].

Histopathological classification traditionally divides sarcomas according to the tissue of apparition. However, sarcomas could also be divided into two main groups according to the type of genetic alterations: (1) sarcomas with near-diploid karyotypes and simple genetic alterations including translocations or specific oncogenic mutations and (2) sarcomas with complex and unbalanced karyotypes. The first group, in turn, could be broken down into three further subgroups: (a) sarcomas carrying translocations, such as SS18-SSX in synovial sarcomas (SS); (b) sarcomas with specific mutated genes, such as c-KIT or platelet-derived growth factor receptor (*PDGFR*) in GIST; and (c) sarcomas with high-level amplifications of chromosome 12 regions encompassing the *MDM2* and *CDK4* loci in well-differentiated liposarcomas (WDLPS) and dedifferentiated liposarcomas (DDLPS). The second group, defined by multiple and unspecific complex karyotype abnormalities, includes loss and amplification of numerous genes, chromosome regions, and chromosomes. To this second group belong leiomyosarcomas (LMS), myxofibrosarcomas (MFS), malignant peripheral nerve sheath tumor (MPNST), pleomorphic liposarcomas (P-LPS), pleomorphic rhabdomyosarcomas (P-RMS), and unclassified pleomorphic sarcoma (UPS) [4]. Since 2002, the differences between these two molecular sarcoma entities have been broadly reviewed in several publications, but the low frequency, the heterogeneity, and the genetic complexity of STS have limited the development of sarcoma genetic profiles. In this line, numerous efforts have been made to establish a gene expression signature in order to improve the prognosis of STS. The first studies, using microarray technology, were based on a small number of samples and a few histologic sarcoma subtypes [5,6]. Since then, other studies have been published describing gene expression related to diagnostic profiles and deregulation of specific signaling pathways. In 2010, Chibon et al. screened the genomic and expression profile of 183 sarcoma tumor samples in order to define a gene expression signature (CINSARC—complexity index in sarcomas) with prognostic value for metastatic events in non-translocation-related sarcomas. The final genetic signature consisted of 67 genes linked to genomic alteration number, tumor aggressiveness, the occurrence of metastasis, and included genes associated with mitosis and chromosome integrity control [7]. Further studies published in 2017 [8] revealed that these CINSARC genes also predicted for other cancer types. In the same year, The Cancer Genome Atlas (TCGA) Research Network reported a broad genomic analysis of 206 frozen adult tumor samples, representing the six major STS subtypes: liposarcomas (LPS), leiomyosarcomas (LMS), undifferentiated pleomorphic sarcoma (UPS), myxofibrosarcoma (MFS), malignant peripheral nerve sheath tumor (MPNST), and synovial sarcoma (SS). In their report, they highlighted two main findings: firstly, sarcomas are mostly characterized by copy number alterations and low somatic mutation burden, whereas only a few genes (*TP53*, *ATRX*, and *RB1*) are repetitively mutated in all sarcoma types, and secondly, molecular subtypes related to patient outcome could be defined based on genomic driver pathways. In addition, they emphasized that the immune microenvironment may have a different impact on patient outcome depending on the sarcoma subtype and might be useful to provide information about immune checkpoint inhibitors in clinical trials [9]. Finally, in 2019, new approaches using transcriptome sequencing data from TCGA revealed that machine learning could be a powerful tool to identify novel diagnostic and prognostic biomarkers [10].

Despite the heterogeneity of these tumors, the first treatment option in patients with localized disease is surgery with or without radiotherapy and chemotherapy [3]. Unfortunately, recurrence is frequent, reaching up to 60% or higher in high-risk localized populations. Doxorubicin (DXR) has been in use for forty years now and still remains the standard first-line treatment for locally advanced unresectable or metastatic STS [11]. Although its combination with ifosfamide was able to extend progression-free survival and improve tumor response rates, it did not achieve significant improvements in terms of overall survival while increasing adverse drug effects [12]. Likewise, although in some randomized trials, treatment with DXR combined with other drugs, such as conatumumab (a monoclonal antibody against TRAIL), palifosfamide (a DNA alkylator), evofosfamide (a hypoxia-activated prodrug, which converts into the alkylating agent bromo-isophosphoramide mustard (Br-IPM)), or olaratumab (an anti-PDGFRA), resulted in a slight benefit in progression-free survival, none of these combinations improved overall survival [13,14,15,16]. Even an alternative combination of different drugs, gemcitabine plus docetaxel, did not produce an advantage in overall survival [17]. These findings clearly show that the search for new molecular drug targets has not ended yet, and that introduction of novel and efficient therapeutic approaches is vital, in particular for patients with metastatic or unresectable tumors, who rely, in the first place, on chemotherapy [18].

The WNT signaling pathway is involved in several fundamental processes of embryogenic development, including the regulation of mesenchymal stem cells, cell migration, and cell turnover to maintain homeostasis in certain adult tissues, such as skin or intestine [19,20]. Traditionally, this signaling pathway is classified as either β-catenin-dependent (canonical) or β-catenin-independent (non-canonical) [21].

The canonical pathway is involved in cell proliferation and survival, while the non-canonical pathway is associated with cell differentiation, migration, and polarity. The non-canonical pathway, in turn, differs in two signaling pathways: the calcium-dependent and the planar cell polarity pathway, and is activated through the binding of WNT ligands to frizzled membrane receptors (FZD) as well as to receptor tyrosine kinase class XII (RYK; related to receptor tyrosine kinase) and class XVII (ROR; receptor tyrosine kinase-like orphan receptors). Despite this theoretical differentiation, both pathways interact to regulate complex processes in a coordinated way, such as embryonic development, maintenance of stem cells, tissue homeostasis, or wound healing, and their aberrant regulation is associated with tumorigenesis, metastasis, and other diseases [22,23].

Although in the literature, the role of the WNT/β-catenin pathway has been widely described in colorectal carcinogenesis as well as in other neoplasms of epithelial origin, little is known about the involvement of this pathway in mesenchymal neoplasms, such as STS. In this review, we first summarize the most relevant molecular alterations of the components of the WNT signaling pathway found in STS and describe their functional roles. The identification of these biomarkers, together with their functional characterization within pathways controlling sarcomagenesis development and progression, is fundamental to improve the current understanding of STS as well as to design new therapeutic approaches for these tumors. The second part of the review describes current therapeutic options modulating WNT signaling and their clinical impact on STS.

## 2. Overview of WNT Signaling Pathway

### 2.1. The Canonical WNT/β-Catenin Pathway

**Figure 1:**
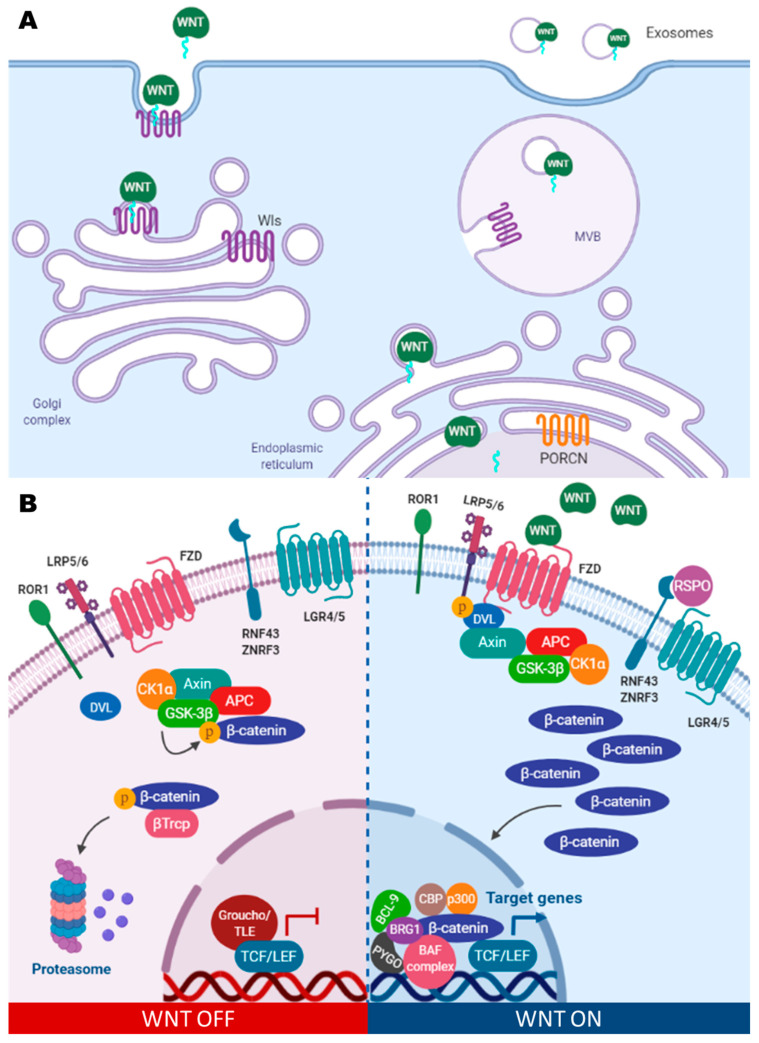
The WNT signaling pathway. (**A**) Scheme of the synthesis and secretion of WNT ligands. At the endoplasmic reticulum, PORCN adds the acyl group palmitic acid to WNT ligands, so that Wls proteins bind to them and transfer them to the plasma membrane. On the right is one of the mechanisms of WNT ligand secretion proposed in the literature, the secretion of WNT ligands through exosomes or extracellular vesicles. (**B**) Scheme of the WNT/β-catenin signaling pathway. On the right, the pathway is activated when WNT ligands bind to FZD and LRP5/6 receptors, activating DVL which inactivates the β-catenin destruction complex formed by Axin, APC, CK1α and GSK-3β. As a result, β-catenin accumulates in the cytoplasm and eventually translocates to the nucleus, where it binds to different co-activators and the TCF/LEF transcription complex to activate transcription of WNT target genes. On the left, in the absence of WNT ligands, the destruction complex phosphorylates β-catenin, which is then recognized by β-Trcp ligase, which marks β-catenin to be degraded at the proteasome.

The WNT ligand family, which is highly evolutionarily conserved [24,25], consists of 19 cysteine-rich glycoproteins that bind to more than 15 receptors or co-receptors [26]. Although several studies have focused on the mechanism by which WNT ligands are produced and secreted outside the cell, there still exist many unknown aspects. During their synthesis, WNT ligands are modified at the endoplasmic reticulum through addition of the acyl group palmitic acid [27,28,29,30] by the enzyme Porcupine (PORCN). Afterwards, Wntless/Evi transmembrane proteins (Wls/Evi) bind to them and transfer them to the plasma membrane, where they are secreted [31,32,33,34,35]. How these secreted WNT ligands reach neighboring cells in order to bind to membrane receptors remains to be determined. Some studies suggest that WNT ligands are transported by extracellular vesicles such as exosomes [36,37,38], so that the WNT ligand located on the surface of these vesicles can bind to the membrane receptor of other cells and activate the pathway. Others propose a model based on direct contact between WNT ligand-producing cells and receptor cells, where frizzled membrane receptors (FZD) and ring finger protein 43 (RNF43)/zinc and ring finger 3 (ZNRF3) transmembrane E3 ligases (see below) play an important role [39,40] (Figure 1A). Although the synthesis of WNT ligands is a common process for all of them, they have historically been classified as canonical (WNT-1, WNT-3A, WNT-8, and WNT-8B) and non-canonical ligands (WNT-4, WNT-5A and WNT-11). The amino-terminal signal sequences determine which WNT signaling will be activated [41]. Nevertheless, another hypothesis suggests that this pathway classification is not entirely correct, since the activation of one pathway or another depends principally on the cellular context and the receptors expressed by cells, rather than on the WNT ligands themselves [35,36].

Activation of the WNT/β-catenin pathway is induced by the binding of extracellular WNT ligands to FZD receptors, which belong to the family of seven-domain transmembrane receptors and their low-density lipoprotein receptor-related protein 5/6 co-receptors (LRP5/6). After the binding of WNT ligands to these receptors, the protein Disheveled (DVL) is phosphorylated and binds to the protein Axin, causing the inhibition of the β-catenin destruction complex, which is composed of several proteins: a structural protein called Axin, adenomatous polyposis coli (APC) enhancing the affinity of the complex for β-catenin, casein kinase 1α (CK1α), as well as glycogen synthase kinase 3β (GSK-3β) (Figure 1B). As a consequence, β-catenin accumulates in the cytoplasm until it translocates into the nucleus, where it binds to the T-cell factor/lymphoid enhancer factor-1 (TCF/LEF) complex, thereby inducing transcription of WNT target genes [42]. Once bound to the TCF/LEF-1 transcription complex, β-catenin binds to a transcriptional co-activator, which can be either cAMP response element binding protein (CREB)-binding protein (CBP) or E1A-binding protein 300 kDa (p300). These two transcriptional co-activators share a degree of homology, for which reason, it has long been suggested that they would have redundant functions. Nevertheless, it has been shown that these two co-activators actually fulfill different roles regarding cell growth and differentiation [43,44,45,46,47]. Thus, it appears that CBP/β-catenin binding initiates the transcription of genes related to the maintenance of pluripotency and stem cell proliferation, whereas p300/β-catenin binding activates a transcriptional program related to cellular differentiation [43,48]. Activation of target pathway genes includes transcription factors such as C-MYC, LEF-1, or TCF, cell cycle regulators such as CCND1 or CDC25A, growth factors such as VEGF or TGF-β, matrix proteins extracellular such as fibronectin, proinflammatory enzymes, and cytokines such as IL-8 or COX-2, as well as proteins such as MMP-2 or MMP-7 [42]. In the absence of WNT ligands, the N-terminus of β-catenin is phosphorylated on serine 45 by CK1α and on threonine 41, serine 33, and serine 37 by GSK-3β, both from the so-called β-catenin destruction complex [49,50,51]. After being phosphorylated, β-catenin is then recognized by the β-Trcp ligase, which labels it to be degraded at the proteasome [49]. Under these conditions, the protein Groucho interacts with the TCF/LEF transcription complex, leading to an inhibition of the transcription of WNT target genes (Figure 1B, left).

### 2.2. The R-Spondin/Lgr5/Rnf43 Axis

In vertebrates, another mechanism of WNT signaling activation has been described, which involves other secreted proteins, the R-spondins (RSPO1–4) [52,53]. When present, RSPOs bind to LGR membrane receptors (Leucine-rich repeat-containing G-protein coupled receptor (LGR4, LGR5 or LGR6), and the resulting complex binds the transmembrane E3 ubiquitin ligases, Ring finger protein 43 (RNF43), and zinc and ring finger 3 (ZNRF3), and prevents them from tagging and removing FZD and LRP6 receptors from the cell surface. As a result, RSPOs increase the presence of WNT receptors on the cell membrane, thereby increasing WNT signaling [54]. In the absence of RSPOs, LGR receptors do not interact with RNF43 and ZNRF3, and the ubiquitin ligases attenuate WNT signaling by promoting ubiquitination and degradation of FZD and LRP6 receptors [54,55,56]. Recent studies have shown that, during this negative regulatory mechanism, DVL protein has a different function from that described above, since it has been linked to the mechanism by which RNF43 and ZNRF3 recognize FZD receptors. In fact, DVL binds to RNF43 and ZNRF3 proteins in order to promote the degradation of FZD receptors [57].

## 3. Deregulation of WNT/β-Catenin Pathway in Soft Tissue Sarcomas

Despite the involvement of the WNT/β-catenin pathway in processes such as self-renewal and differentiation of mesenchymal stem cells, few studies have been published discussing the involvement of the WNT pathway in sarcomagenesis, osteosarcomas (OS), Ewing’s sarcomas (ES), and synovial sarcomas (SS) being the most studied subtypes [58,59,60,61,62]. In 2005, Baird et al. performed an extensive gene expression study in human sarcomas. In this study, 181 sarcoma tumors, taken from frozen tissue of 18 histological different subtypes, were analyzed using high-throughput genetic techniques, and several genes were associated with each sarcoma type, including specific tyrosine kinases, transcription factors, and homeobox genes. Among the top discriminating genes for SS, they found WNT target genes, such as *TLE1*, *WNT-5A*, *FZD1*, and *TLE4*, showing the implication of WNT signaling pathway in this subtype of STS. Moreover, *WNT-5A* and *FZD1* were overexpressed in 11 and 9 of the 16 SS analyzed tumors, respectively [60]. In 2007, Francis et al. reported in a series of 177 high-grade STS a discriminatory gene signature characterized by the overexpression of several genes implicated in the WNT signaling pathway, including *AXIN2*, *LEF1*, *TCF7*, *WISP2*, *FRAG1*, *DAAM1*, *FZD8*, *MYC*, *PRICKLE1*, and *SFRP1* in SS, malignant peripheral nerve sheath tumor (MPNST), and myxoid/round-cell liposarcoma [63]. Table 2 lists the molecular alterations of WNT-signaling pathway components described in STS.

### 3.1. WNT Ligands

WNT ligands consist of 350–400 amino acids and have a molecular weight of about 40 kDa. In order to be secreted, their signal sequences at its amino-terminal end contain hydrophobic amino acids of different lengths that are cleaved for maturation [97]. One of the main WNT ligands activating the canonical WNT signaling cascade is WNT-1. In this context, Chen et al. demonstrated that cells stably expressing WNT-1 are resistant to chemotherapeutic-induced apoptosis, mainly due to their constitutive activation of β-catenin/TCF transcription [98]. In STS, Mikami et al. reported expression of WNT-1 in tissue samples from SS, LPS, and LMS, as well as in metastatic sarcoma cell lines. Moreover, they treated sarcoma cell lines and fresh primary cultures with a monoclonal anti-WNT-1 antibody. The antibody induced cell death and decreased levels of DVL-3 and β-catenin in these sarcoma cells [64]. Later, a case report of a metastatic LMS in the oral cavity highlight that WNT-1 was expressed more in the primary uterine tissue than in metastatic LMS when comparing seven tissue samples [65]. Another WNT ligand, WNT-5A, has been identified as the most significantly upregulated ligand coding gene, and its protein expression has been verified in MPNST when compared to non-tumoral human Schwann cells. However, several functional characteristics of cancer cells, such as uncontrolled growth, capacity to migrate and to invade other tissues, or to promote tumor development (tumorigenicity), were not affected in vitro or in vivo when *WNT-5A* expression was reduced using shRNA in MPNST cells. Rather, *WNT-5A* knockdown increased the expression of genes involved in extracellular matrix remodeling and communication with inmune cells, suggesting that WNT-5A modulates the MPNST microenvironment thereby inhibiting tumor formation [66].

### 3.2. Frizzled Receptors (FZD)

FZD proteins are WNT receptors, which are upregulated in certain tumors. In STS, frizzled homologue 10 (FZD10) is upregulated in SS and plays a role in its cell survival and growth [67]. Moreover, FZD10 is an enhancer of activation of DVL proteins, DVL2/DVL3, and Rac1-JNK cascade, leading to changes in non-canonical signaling and deregulation of actin cytoskeleton [99].

### 3.3. Secreted Frizzled Related Proteins (SFRP)

The naturally secreted WNT pathway inhibitors, such as the secreted frizzled related protein 3 (SFRP3), have been shown to be altered in rhabdomyosarcoma (RMS) expressing the fusion protein PAX3-FOXO1 [70]. By means of shRNA, suppression of *SFRP3* reduced cell growth of human alveolar RMS (A-RMS) cells both in vitro and in vivo. In angiosarcoma (malignant endothelial tumor), another SFRP protein, SFRP2, has been found to be overexpressed. Specific blocking of SFRP2 with a monoclonal antibody in xenograph angiosarcoma mice models and cell lines demonstrated anti-angiogenic effects and inhibition of the β-catenin pathway [68]. Thus, inhibition of SFRPs could represent a novel therapeutic approach in SFRP2-positive STS tumors. However, another member of the SFRP family, SFRP4, was found to be downregulated in a rare uterine tumor, the endometrial stromal sarcoma, as revealed by cDNA arrays [71]. Compared to normal endometrium, the expression of *SFRP4* was decreased in both low-grade endometrial stromal sarcoma and undifferentiated endometrial sarcoma, and it was regulated in an opposite manner to that of β-catenin. Altogether, these data suggest a dual role of these SFRP in the sarcomagenesis of different types of sarcoma.

### 3.4. R-Spondin (RSPOs)

**Figure 2:**
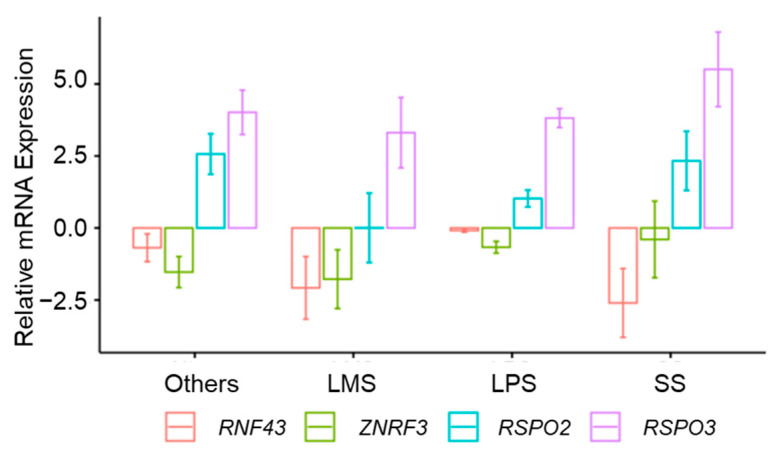
Expression of R-spondin/Lgr5/Rnf43 axis components in tumor samples of STS patients. Values are presented as mRNA expression relative to a pool of RNA of different cell lines (Stratagene QPCR Human Reference Total RNA, 750,500, Agilent Technologies, Santa Clara, California, United States). β-2-microglobulin was used as a reference gene of normalization. Each bar represents the mean ± SEM. Others (*n* = 15), LMS: leiomyosarcoma (*n* = 6), LPS: liposarcoma (*n* = 61) and SS: synovial sarcoma (*n* = 4).

Although RSPOs are unable to initiate WNT signaling [52,54], they potentiate this signaling pathway by increasing the presence of WNT receptors on the cell membrane. Recurrent *RSPO2* and *RSPO3* fusions in colorectal cancer involve *EIF3E* exon 1 and *RSPO2* exon 2 or exon3, and *PTPRK* exon 1 or exon 7 and *RSPO3* exon 3. Interestingly, these *RSPO2* and *RSPO3* gene fusions are mutually exclusive and occurred in tumors without *APC* or *CTNNB1* mutations [100]. In STS, a microarray analysis performed by Watson et al. (2013) revealed that, among the four RSPO proteins, RSPO2 was highly expressed in MPNST when compared with non-tumoral human Schwann cells. Moreover, they determined that the *RSPO2* overexpression was the result of a deletion-mediated gene fusion between exon 1 of *EIF3E* and exon 2 of *RSPO2*, the same as described in colorectal cancer. In vitro, they showed that knockdown of *RSPO2* was able to reduce WNT signaling activity and consequently cell viability. Altogether, their results suggest that overexpression of *RSPO2* could drive a subset of MPNST and that blocking the function of RSPO2 may be a potential therapeutic strategy for MPNST patients with *RSPO2* overexpression [72]. In this line, our group has analyzed *RSPO2* and *RSPO3* mRNA expression in a set of 86 tumor tissue samples representative of different STS subtypes from the University Hospital Son Espases and the Spanish Group for Research on Sarcoma (GEIS). In these STS tissue samples, mRNA levels of *RSPO2* and *RSPO3* were increased 42% and 84%, respectively. *RSPO3* overexpression was a common event in all analyzed STS subtypes; instead, *RSPO2* overexpression was higher in SS compared to LMS and LPS (Figure 2). Nevertheless, a more detailed analysis is needed to confirm RSPO gene fusions in these samples.

### 3.5. Leucine-Rich Repeat-Containing G Protein-Coupled Receptor 5 (LGR5)

Human *LGR5* is a stem cell marker that acts as an oncogene in several human cancers, most notably in colorectal carcinoma. The activation of the membrane receptor LGR5 by RSPO ligands potentiates WNT/β-catenin signaling, contributing to stem cell proliferation and self-renewal. A splice variant of *LGR5* lacking exon 5 was identified in a study carried out with STS patient samples. The presence of low mRNA levels of this transcript variant correlated with poor prognosis in terms of disease-associated survival and recurrence-free survival and thus has been considered a negative prognostic marker in STS. The fact that this variant has a truncated ligand-binding extracellular domain, which modifies its affinity for its ligands, could explain that higher mRNA levels of *LGR5* splice variant are an indicator of better prognosis in STS, possibly due to less activation of canonical WNT signaling in these cases [73]. On the other hand, LGR5 was found to be highly expressed in ES cells and in putative ES cancer stem cells and tumors that display a more aggressive phenotype, suggesting a role of LGR5 in ES tumorigenesis. Thus, in the presence of exogenous WNT and RSPO ligands, LGR5 potentiates WNT/β-catenin signaling in ES cells showing nuclear β-catenin localization and robust activation of TCF reporter activity [101].

### 3.6. Ring Finger Protein 43/Zinc and Ring Finger 3 Transmembrane E3 Ligases (RNF43/ZNRF3)

As mentioned before, RNF43 and ZNRF3 are two homologous transmembrane ubiquitin ligases that induce removal of the FZD and LRP6 receptors from the cell surface via ubiquitin-mediated endocytosis and subsequent lysosomal degradation [54,55,56]. Mutations in *RNF43* and *ZNRF3* have been described widely in different tumors, including colon, pancreas, stomach, ovary, endometrium, and liver cancer [102,103,104,105,106,107,108,109,110,111]. Most common truncating *RNF43* mutations, which are frameshift mutations encoding p.Gly659fs and p.Arg117fs, are associated with microsatellite instability high (MSI-H) phenotype in colorectal and endometrial cancer [108]. In the literature, there is no evidence of *RNF43* or *ZNRF3* mutations in STS, but our group has analyzed mRNA expression of these two ubiquitin ligases in the representative set of STS samples described above. We observed an underexpression of *RNF43* and *ZNRF3* mRNA levels that could be linked to the truncating mutations already mentioned. Among all analyzed STS subtypes, LMS and SS were those with higher underexpression of *RNF43*, while underexpression of *ZNRF3* was similar for all STS subtypes (Figure 2). However, further analyses should be performed to confirm the presence of *RNF43* or *ZNRF3* mutations in these STS samples.

### 3.7. Adenomatous Polyposis Coli (APC)

The APC protein is another key component of the WNT signaling pathway, which acts as a scaffold protein controlling the intracellular levels of the oncoprotein β-catenin. Mutations in *APC* are rare in STS. In fact, so far, only in the study of Tsuyoshi Saito et al. (2002) were 8.2% of SS cases harboring missense *APC* mutations were found. The five discovered mutations, A1299T, G1412R, V1414I, S1398N, and M1413I, were all located in the mutation cluster region (MCR) of the *APC* gene. Moreover, all SS cases with *APC* mutations showed β-catenin accumulation independently of the presence or absence of β-catenin mutation [74].

### 3.8. Glycogen Synthase Kinase 3β (GSK3β)

GSK3β is a signaling mediator of diverse signaling pathways. In the context of WNT signaling, the inhibition of GSK3β-mediated β-catenin phosphorylation is known to be the key event in WNT/β-catenin signaling. In fact, one of the consequences of GSK3β inhibition in cells is the stabilization and nuclear translocation of β-catenin. Some studies have brought to light GSK3β deregulation in STS, mainly in SS. Manish Mani Subramaniam et al. (2010) found expression of phosphorylated GSK3β (active form) in 90 to 100% SS cases studied [75]. In the same trend, a recent study demonstrated that SS and fibrosarcoma (FS) cell lines and patient samples revealed increased activity of GSK3β (higher active form expression), which was responsible for sustained tumor proliferation and invasion. However, in this study GSK3β inhibitors attenuated the growth and invasion of sarcoma cells both in vitro and in vivo without a direct connection to WNT/β-catenin pathway deregulation [76].

### 3.9. β-Catenin (CTNNB1)

β-Catenin is the key transcription factor in the regulation of the canonical WNT signaling cascade. While in many cancers, the aberrant activation of the canonical WNT pathway is due to mutations in this gene or other important components of the pathway such as *APC* [21,112], in the context of STS, few *CTNNB1* mutations have been described. It seems that other mechanisms such as autocrine loop activation [81], fusion proteins [59,93,94], or genomic alterations [60,63] have an important role in the upregulation of the WNT canonical pathway, leading to nuclear β-catenin accumulation and activation of its transcriptional program. Even so, most desmoid tumors (fibrous mesenchymal tumors, including FS) harbor somatic missense mutations in codons 41 (T41A) or 45 (S45F) of exon 3 sequence, which encodes the regulatory degradation targeting box of β-catenin. This allows the mutated protein to escape from the proteasomal degradation and, consequently, to translocate into the nucleus, where it promotes the transcription of WNT target genes [113]. These two different point mutations have also been described in embryonal rhabdomyosarcoma (E-RMS) [77]. Another mutation in *CTNNB1* exon 3 (a C-A transversion at position 37) has been reported as relevant in the oncogenesis of pleomorphic sarcoma. The amino acid change from serine to tyrosine provokes the loss of one of the β-catenin phosphorylation sites, leading to the accumulation of the protein in the cytosol first and then the activation of gene expression by the nuclear β-catenin/TCF4 complex [78]. In addition, *CTNNB1* mutations have also been detected in SS cell lines and in patient samples. The SYO-1 SS cell line carries a point mutation in *CTNNB1* codon 34 (G34L) that does not affect any regulatory phospho-site in the degradation targeting box of β-catenin. Although alterations in this codon do not activate transcription of β-catenin, they cause increased tumor transformation, suggesting that other mechanisms, independently of this mutation, play a crucial role in the activation of canonical WNT signaling [79]. Missense mutations of β-catenin in the regulatory degradation targeting box (T41I and T41A) have been found in a low ratio of SS tumor samples. These mutations do not correlate with nuclear β-catenin expression, as it was found in the majority of these SS tumor samples [75,95]. It is known that SYT-SSX, the oncogenic fusion protein present in most SS, drives constitutively active β-catenin signaling, since it can induce β-catenin nuclear accumulation, apparently by promoting autocrine WNT/β-catenin loop, which is upregulated by aberrant transcriptional effects [82]. This fact may explain that the frequency of nucleocytoplasmic accumulation of β-catenin observed in SS tumors is much higher than the corresponding mutational frequencies found in *CTNNB1*. In addition to SS, many studies have demonstrated a strong nuclear expression of β-catenin in many different STS subtypes in vitro [81,83] and in patients’ tumors [78].

Martinez et al. (2017) demonstrated that, in several STS cells, including stable cell lines and primary cells from patients, β-catenin and phospho-β-catenin (Ser552) were detected, being an *APC*-mutated LMS cell line the one that showed the highest level of active phospho-β-catenin. Moreover, consistent with β-catenin activation, increased basal TCF reporter activity was found in LMS, FS, and LPS cells, and as consequence, *CDC25A*, a WNT target gene that has been described to drive proliferation of sarcomas [81], was overexpressed in STS cells. In summary, these results corroborate that WNT signaling is upregulated in STS cells.

### 3.10. Transducin-Like Enhancer 1 (TLE-1)

TLE-1 is a transcriptional co-repressor that inhibits the WNT signaling pathway and other cell fate determination signaling pathways. It has no-DNA binding domains but can promote specific gene silencing by downregulating transcriptional activators by enhancing transcriptional repressors or by transforming transcriptional activators into repressors. Generally, TLE-1 inhibits WNT signaling transcription through its binding to TCF/LEF. In SS, the association between the SS18-SSX fusion protein and overexpression of TLE-1 has been described. The concrete mechanism of action of TLE-1 in SS consists of downregulating the activating transcription factor 2 (ATF2). In non-tumoral cells, ATF2 enhances the transcription of early growth response 1 (EGR1) target genes, involving tumor suppressors such as TP53 and PTEN. In SS, the SS18-SSX fusion protein forms a stable complex with TLE-1 and ATF2 in the nucleus that, in turn, represses the transcriptional activity of EGR1 and boosts oncogenesis [59,101,102]. High protein levels of TLE-1 in the nucleus are a characteristic feature of SS, thus making it a differential diagnostic marker between SS and histologically similar tumors, such as MPNST. In fact, nuclear TLE-1 detection by immunohistochemistry is accepted as a diagnostic tool for SS in clinical practice [59,85,86,87,92].

### 3.11. cAMP Response Element-Binding Protein (CREB)-Binding Protein, CBP (CREBBP)

CBP is one of the transcriptional co-activators of canonical WNT signaling, and it exerts its effects when interacting with β-catenin in the nucleus. *CREBBP* gene mutations have been found in histiocytic sarcomas, which are rare, and aggressive neoplasms of mature histiocytes that can arise de novo or as a secondary malignancy evolving from low-grade B cell lymphoma. The possible common origin between these two neoplasias highlights the need for an accurate differential diagnosis. The detection of somatic mutations I1471T and F1484V in the histone acetyltransferase domain of CREBBP may help with the differential diagnosis [88]. These mutations are associated with functional activation of BCL6, a transcriptional co-repressor of WNT signaling. Thus, some studies have pointed out that BCL6 downregulates proliferative pathways, such as the WNT pathway, but also upregulates a neurogenic differentiation program, which makes sense in the context of histiocytic sarcoma development [114]. Some sarcomas can also arise in the setting of a recognized heritable cancer predisposition syndrome, and that can be the case of E-RMS or LMS, which are associated with Rubinstein–Taybi syndrome patients harboring the *CREBBP* mutation at 16p13.3. In fact, mutations of *CREBBP* gene have been reported in approximately half of Rubinstein–Taybi syndrome patients, which would have the potential to develop E-RMS or LMS. Identification of such correlations, if present, can facilitate appropriate genetic counseling and testing of patients and their relatives, as well as screening, surveillance, and interventional measures, as needed [89,90].

### 3.12. Transcription Activator BRG-1 (SMARCA4)

The protein BRG-1, a subunit of the SWI/SNF chromatin-remodeling protein complex (BAF), is encoded by the *SMARCA4* gene and modulates WNT target gene expression through the binding to β-catenin. It is considered a tumor suppressor gene, because many loss-of-function alterations in this subunit are increasingly detected in human malignancies. Cancer-related *SMARCA4* mutations alter the open chromatin landscape promoting the expression of pro-oncogenic genes [115]. In the last five years, the detection of inactivating mutations in *SMARCA4* that cause loss of nuclear protein expression has emerged as a differential diagnostic marker between some types of sarcoma and carcinomas sharing similar molecular characteristics. For example, in combination with a panel of other molecular features, SMARCA4 has become a novel tool to discern between SMARCA4-deficient undifferentiated uterine sarcoma and undifferentiated and dedifferentiated endometrial carcinomas (generally expressing intact SMARCA4). Kolin et al. (2020) found a wide variety of *SMARCA4* inactivating mutations among the 12 cases of undifferentiated uterine sarcomas studied: one loss-of-function inversion and different frameshift, nonsense, missense, and splice site mutations. One of the cases also presented loss of heterozygosity (LOH) [91]. Following this approach, and taking into account *SMARCA4* mutational studies and copy number analysis, a new molecular entity of aggressive tumors has been described. These studies have provided evidence of consistent alteration at 19p13 encompassing *SMARCA4* in thoracic sarcomas, resulting in either LOH or heterozygous deletions. The thoracic sarcomas carrying inactivation mutations in *SMARCA4* are enriched in genes involved in proliferation and stemness compared to unclassified sarcomas. Thus, SMARCA4-deficient thoracic sarcomas are at present recognizable in clinical practice, due to simultaneous loss of expression of *SMARCA4* and *SMARCA2* and concomitant overexpression of *SOX2*. In this case, the immunodetection of SMARCA4 serves to distinguish between thoracic sarcomas and lung carcinomas, as the latter usually do not display a loss of SMARCA4 nuclear expression [106,113,114]. Another example of mutation in *SMARCA4* that has been associated with an increased risk for developing a malignant type of soft tissue tumor called rhabdoid tumor is that germline heterozygous loss-of-function mutations in *SMARCA4* (19q13.2) cause rhabdoid tumor predisposition syndrome II. As mentioned before, the identification of carriers involved in the development of malignant STS is an important fact to take into account, because it allows adequate monitoring and management of patients [90].

### 3.13. E1A Binding Protein 300 kDa, p300 (EP300)

p300 is a member of the histone acetyltransferase family of transcriptional co-activators that can bind to β-catenin when it accumulates in the nucleus due to WNT signaling activation. In a study of 43 SS tumor samples, Liu et al. (2019) found high p300 protein expression in SS, being more prevalent in monophasic SS. Although high p300 expression has been associated with poor overall survival (OS), progression-free survival, and recurrence-free survival in various types of human cancer, such as digestive system malignant neoplasms, the prognostic value of p300 expression has not been proved yet in SS [94].

### 3.14. Lymphoid Enhancer-Binding Factor 1 (LEF1)

*LEF1* gene encoding for the transcription factor lymphoid enhancer-binding factor 1 participates in the WNT signaling pathway, activating the transcription of target genes in the presence of β-catenin and its co-activators. LEF1 is altered in most SS, which display high mRNA levels and strong nuclear expression, providing evidence that aberrant activation of the WNT/β-catenin pathway is present in SS [95]. On the other hand, Dräger et al. (2017) reported a subset of A-RMS and E-RMS tumors overexpressing LEF1 compared to normal skeletal muscle. Contrary to the expected WNT transcriptional promoting effects of LEF1, knockdown of this gene in in vitro and in vivo models of A-RMS and E-RMS generally resulted in increased proliferation, migration, and invasiveness, accompanied by inhibition of apoptosis. These observations suggest a rather suppressive role of LEF1 on canonical WNT signaling in the two major subtypes of RMS: A-RMS and E-RMS [96].

## 4. Targeting WNT Signaling Pathway in STS

The molecular alterations described above are potential drug targets for the development of new therapies for STS that aim at key molecular members of the WNT signaling pathway.

**Figure 3:**
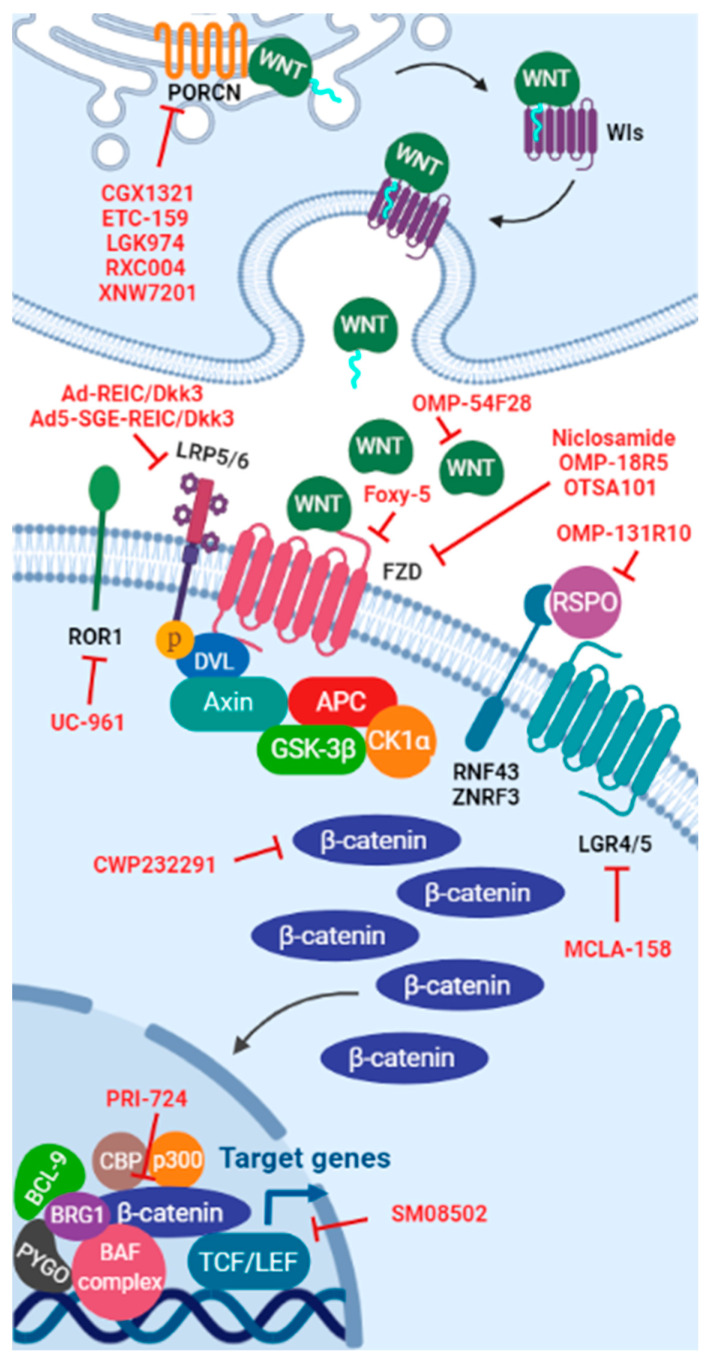
WNT signaling pathway inhibitors. Scheme of the WNT signaling pathway showing the interaction site of each WNT inhibitor include in clinical trials.

Specific WNT signaling pathway inhibitors can be classified into functional groups according to their interaction site: upstream of β-catenin or downstream of β-catenin (Figure 3). The molecular mechanisms behind upstream of β-catenin inhibitors evaluated in clinical trials include: (a) inhibition of WNT ligands secretion, (b) disruption of WNT ligand-receptor interaction, and (c) induction of β-catenin degradation by activation of caspase-3-dependent apoptosis. Furthermore, strategies involved in WNT signaling inhibition downstream of β-catenin include: (a) disruption of β-catenin interaction with its co-activators in the nucleus and (b) inhibition of WNT target genes and their protein levels by alternative mechanisms, mainly reducing spliceosome activity via CDC-like kinase (CLK) inhibition.

Of the 44 clinical trials focusing on targeting WNT signaling pathway on cancer treatment, 39 trials (89%) have as a target WNT-upstream members, while only 5 of them (11%) focus on pathway inhibition downstream of β-catenin (Table 3).

### 4.1. Therapeutic Strategies Upstream of β-Catenin

#### 4.1.1. Inhibition of WNT Ligands Secretion

Porcupine (PORCN) is considered a highly selective target for the WNT driven cancers [116], as this protein has no other biological function than to play a central role in the secretion and activity of WNT ligands [40,117] (Figure 1A). PORCN inhibitors suppress the WNT signaling pathway by inhibition of the biogenesis of WNT ligands through preventing their palmitoylation. Preclinical studies have highlighted that tumors with upstream aberrations in the WNT signaling pathway (*RNF43* and *ZNRF3* loss-of-function mutations, RSPOs translocations, and Notch ligand loss-of-function mutations) are WNT ligand dependent, and those aberrations have a huge potential to be used as biomarkers to predict sensitivity to PORCN inhibitors [118,119,120,121,122]. Several PORCN inhibitors have already been synthesized and have demonstrated their efficacy and good tolerance in preclinical studies. Furthermore some PORCN inhibitors are currently being explored in different clinical trials (Table 4). Among them, five molecules have reached phase I/II: ETC-159 [123], XNW7201 [124] and RXC004 [125] for advanced metastatic solid tumors, CGX1321 [126,127] also for gastrointestinal tumors, and LGK974 for malignancies dependent on WNT ligands [128,129,130].

ETC-159 has been reported to be highly efficacious in genetically defined colorectal cancer tumors with *RSPO2/3* translocations [122] and was proven to be safe and well-tolerated as a single agent in an initial phase I trial [167]. Later on, in a second part of this trial, predicted to conclude in 2023, ETC-159 will be administrated in combination with the immunomodulatory monoclonal antibody pembrolizumab.

RXC004 in vitro reduced cell proliferation through cell cycle arrest at G1/S and G2/M, in colorectal and pancreatic cell lines harboring *RNF43* mutations and *RSPOs* fusions. Its effects were accompanied by a significant reduction in mRNA expression of WNT targets as *AXIN2* and *C-MYC* [168]. Oral treatment with RXC004 in xenograft models for pancreatic and gastric cancer (with *RNF43* mutations) also exhibited a potent inhibition of tumor growth [169]. Currently, patients with advanced malignancies are being recruited in a phase I/II trial to treat them with RXC004 alone or in combination with other anticancer drugs.

CGX1321 is a potent and specific inhibitor developed through several in silico approaches in a comprehensive study from a lead compound series (patent: US9556144) [170,171]. Studies in vivo [172] showed that CGX1321 strongly reduced tumor growth in PDX mouse models harboring *RSPO2* fusions after 28 days of treatment. Nowadays, this inhibitor is in phase I trials for treatment of patients with advanced solid tumors or with advanced gastrointestinal tumors as a single agent or in combination with pembrolizumab.

LGK974, also known as WNT974, is an oral agent under investigation that potently inhibits PORCN. Preclinical studies pointed out that cancers with mutations in *RNF43* or *ZNRF3*, and fusions in RSPOs are predicted to be sensitive to this inhibitor. Moreover, preliminary clinical data shows that LGK974 has promising potential for antitumor activity in selected patients harboring WNT aberrations [173]. At this moment, of all the above-mentioned PORCN inhibitors, only LGK974 has exhibited an antitumoral effect in Ewing sarcoma (ES). Hayashi et al. (2017) demonstrated that LGK974 suppressed the expression of genes involved in motility, invasion, and metastasis without affecting tumor cell proliferation in vitro or tumor growth in vivo (patient-derived xenografts) [174]. Although our own study did not test the presence of mutations in *RNF43* and/or *ZNRF3* or of fusions that provoke the amplification of *RSPO2* and/or *RSPO3*, these genes were clearly under- and overexpressed in STS cell lines and in patients’ tumors (Figure 2). Therefore, it can be speculated that some STS, and especially a subset of MPNST with *RSPO2* overexpression, might respond to PORCN inhibitors.

#### 4.1.2. Disruption of WNT Ligand–Receptor Interaction

Other upstream inhibitors are focused on disrupting the interaction of WNT ligands and their receptors instead of preventing the synthesis of the ligands. These inhibitors include small molecules and monoclonal antibodies targeting both WNT ligands and WNT receptors. Due to redundancy among WNT ligands, more attempts have been made to target WNT receptors. In line with this, among the inhibitors that have reached phase I/II clinical trials, we identify three molecules targeting WNT ligands, Foxy-5 [131,132,133], OMP-131R10 (Rosmantuzumab) [138], and OMP-54F28 (Ipafricept) [134,135,136,137], and six molecules targeting WNT receptors, Ad5-SGE-REIC/Dkk3 [139,140], MCLA-158 [141], Niclosamide [142,143,144,145,146,147], OMP18R5 (Vantictumab) [148,149,150,151], OTSA101 [152,153], and UC-961 (Cirmtuzumab) [154,155,156,157,158] (Table 4).

Molecules targeting WNT ligands include Foxy-5, a formylated WNT-5A-derived hexapeptide that has demonstrated its potential to mimic the effects produced by the full WNT-5A ligand. In this context, Foxy-5 was able to impair the motility of breast cancer cell lines with low levels of endogenous WNT-5A [175] and to reduce the early metastatic spread of WNT-5A-low prostate cancer cells in an orthotopic mouse model [176]. Consequently, the compound reached phase I clinical trials as a single agent for metastatic breast and prostate cancer in 2013 and 2016 [131,132]. Currently, metastatic colorectal cancer patients are being recruited and randomized to receive Foxy-5 as a single agent or as a neo-adjuvant administration in a phase II clinical trial [133], which is expected to conclude on 2022.

The molecule OMP-131R10 (Rosmantuzumab) is an anti-RSPO3 monoclonal antibody that entered clinical trials in 2015 as a single agent for advanced solid tumors and in combination with chemotherapy (FOLFIRI), including metastatic colorectal cancer [138]. The identification of *RSPO3* overexpression in STS patients’ tissue samples by our group could be used as preliminary data to support further preclinical studies in order to evaluate the effect of this monoclonal antibody in STS, whether administrated alone or in combination with DXR.

Another molecule that binds WNT ligands is OMP-54F28 (Ipafricept) [137], a recombinant fusion protein with the extracellular part of the human FZD8 receptor fused to a human IgG1 Fc fragment, which can bind WNT ligands. Despite not showing marked responses in patients with advanced solid tumors treated with Ipafricept as a single agent in a phase I clinical trial [177] (of 26 patients, only two desmoid tumor and one germ cell cancer patients presented stable disease for > 6 months), three phase Ib studies assessing Ipafricept in combination with several chemotherapy agents, such as sorafenib for hepatocellular cancer, paclitaxel and carboplatin for ovarian cancer, and Nab-paclitaxel and gemcitabine for pancreatic cancer [178,179,180] have been completed. In this last study, 34.6% of patients had partial response and 46.2% stable disease, demonstrating a clinical benefit rate of 81% [178]. In all the studies, Ipafricept was well tolerated, but bone toxicity at efficacy doses has limited further development of this inhibitor. Nevertheless, different approaches, including patient selection, monitoring, zoledronic acid administration, and modification of the dose and schedule, have been implemented to mitigate this adverse drug reaction [170,172,174].

Clinical trials using WNT receptor inhibitors include Ad5-SGE-REIC/Dkk3 [139,140] for prostate cancer and MCLA-158 [152] for metastatic colorectal cancer. Niclosamide [151,152,154] entered a phase I clinical trial for patients with colon cancer that were undergoing primary resection of their tumor in 2016. However, this clinical trial stopped early due to low accrual. Even so, a phase II clinical trial with this drug began in 2015 to investigate its safety and efficacy when administrated orally in patients with colorectal cancer progression after therapy. Later on, a phase II clinical trial started in 2020 to evaluate its effect on colorectum and duodenum polyps in familial adenomatous polyposis (FAP) patients. This clinical trial is currently recruiting patients and aims to be completed in 2023. Some phase I clinical trials with niclosamide started in patients with castration resistant prostate cancer (CRPC) in 2015 and 2017 [144,147]. The objective of these trials was to evaluate niclosamide side effects and its adequate doses when given together with enzalutamide, an androgen receptor inhibitor. Another phase II clinical trial with this drug started in 2016 but is still recruiting CRPC patients to evaluate its side effects when administrated in combination with abiraterone (an anti-androgen drug).

OMP18R5 (Vantictumab) [149,150,151] is a fully human monoclonal antibody that inhibits WNT pathway signaling through binding Frizzled receptors 1, 2, 5, 7, and 8. In several clinical trials, it has been combined with docetaxel for non-small cell lung cancer (NSCLC) treatment, with paclitaxel for locally recurrent or metastatic breast cancer treatment, and with Nab-paclitaxel or gemcitabine in pancreatic cancer treatment.

Another molecule, UC-961 (Cirmtuzumab) [154,156,157,158], has been studied in patients with chronic lymphocytic leukemia (CLL) as a single agent or in combination with venetoclax (an inhibitor of BCL-2). It has also been administered to patients with HER2-negative breast cancer in combination with paclitaxel and given as a single agent or in combination with ibrutinib (a tyrosine kinase inhibitor) to patients with B-cell lymphoid malignancies.

The FZD10 receptor has been reported to be highly expressed on the cell surface of almost all SS tumors, but it is absent in most healthy organs except for the placenta [59,181]. As a consequence, antibodies against FZD10 have shown a therapeutic effect for patients with SS tumors overexpressing FZD10. The anti-tumor effects of these antibodies is enhanced when they are radiolabelled [181], while showing minimal or no adverse reactions, since FZD10 protein is hardly expressed in healthy organs [67,182]. OTSA101, a chimeric humanized monoclonal antibody against FZD10 receptor [152,153], slightly inhibited cell growth in several SS cell lines when administered as non-radiolabelled in preclinical studies. Despite this, in vivo studies performed with Yttrium 90-radiolabeled OTSA101 showed significant anti-tumor activity in mouse xenograft models [59,175,177]. These results led to initiate a phase I clinical trial in 2011 administering Yttrium 90-radiolabeled OTSA101 to treat SS patients, which were resistant to DXR and ifosfamide [153]. Unfortunately, this study stopped in 2015 due too slow accrual. Additionally, a new phase I clinical trial is currently recruiting patients with relapsed or refractory SS to be treated with Yttrium 90-radiolabeled OTSA101 and is expected to conclude in 2022 [152].

#### 4.1.3. Induction of β-Catenin Degradation

CWP232291 is a peptidomimetic small molecule that induces degradation of β-catenin through endoplasmic reticulum (ER) stress activation. ER stress leads to the activation of caspase-3-mediated apoptosis, which can induce degradation of β-catenin [183,184], and thereby inhibits cell cycle progression and anti-apoptotic WNT target genes, such as cyclin D1 and survivin [185]. Preclinical studies have shown significant anti-tumor activity of CWP232291 in vitro and in vivo models for acute myeloid and multiple leukemia [186,187] and for CRPC [185] (Table 4). In 2011 and 2015, the inhibitor reached phase I clinical trials for acute myeloid leukemia and multiple myeloma [159,161], showing modest overall efficacy as a single agent and leading to its use in combination with other antitumor agents [188]. It is currently being assessed in phase I/II clinical trials for relapsed or refractory acute myeloid leukemia patients in combination with ara-C or cytarabine [160]. In STS, it would be interesting to evaluate the use of this small molecule in those FS, E-RMS, or pleomorphic sarcoma tumors harboring somatic missense mutations in *CTNNB1* that allow mutated β-catenin to escape from proteasomal degradation, as the mechanism of induction of β-catenin degradation behind CWP232291 is completely different.

### 4.2. Therapeutic Strategies Downstream of β-Catenin

#### 4.2.1. Disruption of β-Catenin Interaction with Its Co-Activators

After translocation into the nucleus, β-catenin activates transcription of WNT target genes by recruiting a transcriptional co-activator, which can be either cAMP-response-element-binding protein (CREB)-binding protein (CBP) or E1A binding protein 300 kDa (p300). Several high-throughput screenings have been conducted to identify small molecules targeting these interactions, as this has been considered a promising therapeutic approach. Among them, ICG-001, a first-in-class inhibitor of β-catenin/CBP interaction, was developed in 2004. In preclinical studies, this inhibitor has been reported to reduce cell viability and to induce apoptosis in LPS cells [189] and colorectal carcinoma cells [190,191]. In triple negative breast cancer (TNCB), ICG-001 was also effective preventing tumor growth and metastasis in a highly metastatic chemoresistant patient-derived xenograft model. Interestingly, the combination of this inhibitor with the chemotherapeutic agent DXR demonstrated a synergistic action in vivo, suggesting that the addition of ICG-001 to a DXR regimen would be efficacious in patients with TNBC [192]. Follow-up studies suggested this inhibitor as a potential second-line therapy in TNBC patients who progress after anthracycline treatment [193]. Its derivative, PRI-724, has reached phase I/II clinical trials for advanced solid tumors, advanced myeloid malignancies, and advanced pancreatic cancer [163,164,165] (Table 4). PRI-724 selectively binds to CBP, impairing its interaction with β-catenin, thereby inhibiting the expression of WNT target genes induced by this interaction. PRI-724 is highly selective for CBP and does not interact with the other co-activator, p300. Furthermore, in preclinical studies, PRI-724 increased β-catenin/p300 interaction and promoted stem cell differentiation, thereby increasing sensitivity to cytotoxic or targeted inhibitors. In clinical trials, PRI-724 has demonstrated a safe toxicity profile when administrated as a single agent in patients with advanced solid tumors [194]. Moreover, PRI-724 demonstrated a modest anti-tumoral activity in combination with gemcitabine as second line therapy in patients with advanced pancreatic cancer [195].

In STS, the effect of PRI-724 has been very recently evaluated by our group (Martinez et al., 2020). The study demonstrated that PRI-724 significantly reduces cell proliferation of different subtypes of STS cell lines in vitro by downregulating the expression of the WNT target gene *CDC25A*, which is highly expressed in STS patient samples, thereby inducing cell death or cycle arrest. Most importantly, combination of PRI-724 with standard STS chemotherapeutic drugs, DXR or trabectedin, enhanced their antitumor activity in a synergistic manner, suggesting that this new strategy with PRI-724 could also have a beneficial clinical effect on patients with STS [196]. These results are a basis for future clinical trials evaluating inhibitors of β-catenin–co-activator interactions in STS patients with an activated Wnt signaling pathway, the latter being a novel selection criterion that could be included when recruiting patients.

#### 4.2.2. Inhibition of WNT Target Genes and Their Protein Levels by Alternative Mechanisms, Mainly Reducing Spliceosome Activity

The expression of WNT target genes could also be reduced by alternative mechanisms, such as modulating the alternative splicing. SM08502 is an inhibitor of CDC-like kinase (CLK), which has an important role in precursor-mRNA splicing [197,198,199,200], and a decrease in spliceosome activity leads to downregulation of WNT target genes. Tam et al. (2020) reported that in colorectal cancer cells, SM08502 inhibited the expression of WNT target genes in vitro (*AXIN2*, *LEF1*, *MYC*, and *TCF7*) more than 10-fold more than PRI-724, which also acts downstream of β-catenin [200]. Moreover, in xenograft mouse models, they found a significant reduction in gastrointestinal tumor growth due to downregulation of WNT target gene expression (*TCF7*, *MYC*, *LRP5*, *DVL2*, and *BTRC*). Currently, patients with advanced solid tumors are being recruited in a phase I clinical trial to evaluate the safety and pharmacokinetics of this inhibitor when administered orally [166] (Table 4). The revelation of the important role of the WNT target gene *CDC25A* in STS [92,100,197] provides evidence that alternative mechanisms inhibiting WNT target gene expression might also be assessed in patients with STS tumors. In line with this hypothesis, SM09419, another CLK inhibitor, has also demonstrated a potent inhibition of CLK and the WNT signaling pathway in preclinical studies. This inhibitor suppressed the expression of WNT target genes (*CCND1*, *LEF1* and *TCF7*) in mantle cell lymphoma (MCL) and strongly impaired cell proliferation and induced apoptosis in vitro in MCL and in acute myeloid leukemia (AML) cell lines. In vivo, this compound demonstrated a strong antitumor effect in both neoplasias, and consequently, a phase 1 study assessing safety, tolerability, and pharmacokinetics of SM09419 in subjects with advanced hematologic malignancies has already been initiated [201,202].

### 4.3. Upcoming Therapeutic Strategies

Even though WNT signaling was implicated in the immune modulation more than one decade ago [203,204], only recently have more detailed correlations between WNT and cancer immunosurveillance been elucidated. An analysis of various human cancer types using TCGA revealed that 33.9% were non-T-cell-inflamed tumors. Interestingly, mutations of components of canonical WNT signaling were three-fold higher in this subset [205]. Melanoma is one of the cancer types where immunotherapy has shown better clinical responses. However, not all patients respond to the same extent to this therapy, and the mechanisms underlying those differences are broadly unknown. Active Wnt/β-catenin signaling in melanoma promotes exclusion of T-cells and resistance to anti-PD-L1/anti-CTLA-4 therapy. These data suggest that this signaling pathway is able to influence the tumor microenvironment, leading to immunotherapy resistance [206]. Additionally, melanoma cells can activate WNT signaling in a paracrine way in dendritic cells, thereby preventing immunoreactivity through indoleamine 2,3-dioxigenase 1 (IDO-1) [207]. In sarcomas, IDO-1 targeting has been addressed in a publication where the IDO-1 expression was significantly correlated with high CD8+ infiltration, but no antitumoral activity was found with the combination of IDO inhibitor plus PD-L1 blockade [208]. In correlative studies of phase II trials in sarcoma, testing the impact of PD1/PD-L1 inhibition, and in recent reviews, no relevant correlation implying WNT signaling in poor responders has been reported [209,210,211,212,213]. However, the number of enrolled patients was limited, and this correlation has not been specially explored yet. Knowing the relevance of WNT signaling in the sarcoma context, and taking into account that sarcomas exhibit a cold microenvironment, it will be important to focus future research on the combined strategies of WNT signaling inhibition and immunomodulation treatment in each specific sarcoma subtype.

## 5. Conclusions

This review summarizes the current knowledge about the WNT/β-catenin pathway and its role in tumoral processes. A growing body of evidence shows that deregulation of canonical as well as non-canonical WNT signaling is an essential event during oncogenesis and uncontrolled cell proliferation in a variety of STS subtypes, which are tumors of mesenchymal origin. The latter is consistent with the fact that the WNT/β-catenin pathway is also involved in pivotal physiological processes (for example, self-renewal and differentiation) of mesenchymal stem cells, which, by its very nature, have unlimited potential to proliferate. It can be hypothesized that STS patients might benefit from both upstream inhibitors of β-catenin, such as PORCN inhibitors or WNT ligand-receptor interaction inhibitors, as well as downstream inhibitors of β-catenin, both of which have shown promising results in clinical treatments of neoplasms of epithelial origin. Although only few clinical trials with WNT/β-catenin pathway inhibitors have been conducted with STS patients, the molecular data suggest that especially those patients with an over-activated WNT signaling pathway could benefit from its inhibition. In this context, determination of the specific deregulation sites in every patient and the development of novel, specifically targeted drugs, administrated as single agents or in combination with other chemotherapeutics, would be an important step towards precision medicine in STS treatment.

## Figures and Tables

**Table 1:** Summary of the WHO histological classification of STS.

Subgroup	Malignant (e.g.,)
Adipocytic tumors	Liposarcoma
Fibroblastic and myofibroblastic tumors	Myxofibrosarcoma
So-called fibrohistiocytic tumors	Malignant tenosynovial giant cell tumor
Vascular tumors	Angiosarcoma
Pericytic (perivascular) tumors	Glomus tumor
Smooth muscle tumors	Leiomyosarcoma
Skeletal muscle tumors	Rhabdomyosarcoma
Gastrointestinal stromal tumors	Gastrointestinal stromal tumor
Chondro-osseous tumors	Osteosarcoma extraskeletal
Peripheral nerve sheath tumors	Malignant peripheral nerve sheath tumor
Tumors of uncertain differentiation	Synovial sarcoma

**Table 2:** Molecular alterations of WNT signaling pathway components in STS.

Wnt Pathway Component	Alteration	STS Subtype	Effect on WNT Signaling or Tumoral Phenotype If Known	Reference
WNT-1	High protein expression	SS, LPS, and LMS	Involved in metastatic phenotype	[64,65]
WNT-5A	Upregulated mRNA and protein expression	MPNST	Modulation of tumoral microenvironment	[66]
FZD10	Upregulated mRNA and protein expression	SS	Involvement in tumor growth	[67]
SFRP2	High protein expression	Angiosarcoma	Non-canonical WNT pathway activation	[68,69]
SFRP3	Upregulated mRNA expression	A-RMS	Involvement in tumor growth	[70]
SFRP4	Downregulated mRNA expression	Endometrial stromal sarcoma	Allows high β-catenin expression (inverse correlation)	[71]
RSPO2	Upregulated mRNA expression	STS, MPNST	Driver of WNT signaling and cell growth	[72]
RSPO3	Upregulated mRNA expression	STS	Unknown	
LGR5	Splice variant	STS	Correlation between low mRNA and a higher risk of tumor-related death	[73]
RNF43	Downregulated mRNA expression	STS	Unknown	
ZNRF3	Downregulated mRNA expression	STS	Unknown	
APC	Missense mutations	SS	Uncertain contribution to nuclear β-catenin accumulation and activation of canonical WNT signaling	[74]
GSK3β	High protein expression	SS and FS	Involvement in tumor growth without an effect on the WNT signaling	[75,76]
CTNNB1	Missense mutations	E-RMS	Unknown	[77]
	Missense mutation	Pleomorphic sarcoma	Nuclear β-catenin accumulation and transcription of WNT target genes	[78]
	Missense mutations	SS	Uncertain contribution to nuclear β-catenin accumulation and activation of canonical WNT signaling	[75,79,80]
	High nuclear expression	Many STS	Transcriptional activation of WNT target genes causing high proliferative activity	[78,81,82,83]
TLE-1	High nuclear expression	SS	TLE-1/SS18-SSX/ATF2 complex represses EGR1 transcriptional activity, silencing tumor suppressor genes	[84,85,86,87]
CREBBP	Missense mutations	Histiocytic sarcoma	Unknown	[88]
	Germline mutation ^1^	E-RMS and LMS	Tumor development potential	[89,90]
SMARCA4	Inactivating mutations *	Undifferentiated uterine sarcoma	Unknown	[91]
	Inactivating mutations **	Thoracic sarcoma	Upregulation of proliferation and stemness genes associated with loss of nuclear protein expression	[92,93]
	Germline mutation ^2^	Malignant rhabdoid tumors	Tumor development potential	[90]
EP300	High nuclear expression	SS	Unknown	[94]
LEF1	High mRNA and nuclear expression	SS	Aberrant activation of canonical WNT signaling	[95]
	High nuclear expression	A-RMS and E-RMS	Tumoral progression attenuation and induction of myodifferentiation	[96]

^1^ found in Rubinstein-Taybi syndrome patients. ^2^ found in Rhabdoid tumor predisposition syndrome II patients. * SNVs and rearrangements. ** SNVs and indels.

**Table 3:** Different therapeutic strategies performed in clinical trials to inhibit the WNT signaling pathway.

Mechanism of Action	Number of Clinical Trials
Upstream of β-catenin	39
Inhibition of WNT secretion	8
WNT ligand-receptor interactions	28
β-catenin degradation	3
Downstream of β-catenin	5
β-catenin—co-activator interactions	4
Alternative mechanism	1
Total	44

**Table 4:** Cancer related clinical trials involving WNT signaling pathway inhibitors.

Mechanism of Action	Inhibitor	Clinical Trial	Phase	Cancer Type	Reference
Porcupine inhibition	CGX1321	NCT03507998	I	Colorectal/Gastric/Pancreatic Adenocarcinoma, Bile Duct/Hepatocellular/Esophageal Carcinoma, Gastrointestinal Cancer	[126]
		NCT02675946	I	Solid Tumors, Gastrointestinal Cancer	[127]
	ETC-159 (ETC-1922159)	NCT02521844	I	Solid Tumors	[123]
	LGK974 (WNT974)	NCT01351103	I	Pancreatic/*BRAF* Mutant Colorectal/Triple Negative Breast/Head and Neck Squamous Cell/Cervical Squamous Cell/Esophageal Squamous Cell/Lung Squamous Cell Cancer, Melanoma	[128]
		NCT02649530	II	Squamous Cell Carcinoma, Head and Neck	[129]
		NCT02278133	I|II	Metastatic Colorectal Cancer	[130]
	RXC004	NCT03447470	I	Cancer, Solid Tumors	[125]
	XNW7201	NCT03901950	I	Advanced Solid Tumors	[124]
WNT-5A—Frizzled-5 interaction	Foxy-5	NCT02020291	I	Metastatic Breast/Colorectal/Prostate Cancer	[131]
		NCT02655952	I	Metastatic Breast/Metastatic Colon/Metastatic Prostate Cancer	[132]
		NCT03883802	II	Colon Cancer	[133]
WNT ligands	OMP-54F28 (Ipafricept)	NCT02050178	I	Pancreatic/Stage IV Pancreatic Cancer	[134]
		NCT02069145	I	Hepatocellular/Liver Cancer	[135]
		NCT02092363	I	Ovarian Cancer	[136]
		NCT01608867	I	Solid Tumors	[137]
RSPO3	OMP-131R10 (rosmantuzumab)	NCT02482441	I	Advanced Relapsed/Refractory Solid Tumors	[138]
LRP5/6 receptors	Ad-REIC/Dkk3	NCT01197209	I	Prostate Cancer	[139]
	Ad5-SGE-REIC/ Dkk3	NCT01931046	I|II	Localized Prostate Cancer	[140]
EGFR/LGR5 receptors	MCLA-158	NCT03526835	I	Advanced/Metastatic Solid Tumors, Colorectal Cancer	[141]
Frizzled receptors	Niclosamide	NCT02687009	I	Colon Cancer	[142]
		NCT04296851	II	Familial Adenomatous Polyposis	[143]
		NCT02532114	I	Castration Levels of Testosterone, Castration-Resistant Prostate/Metastatic Prostate/Recurrent Prostate Carcinoma, Stage IV Prostate Adenocarcinoma	[144]
		NCT02519582	II	Colorectal Cancer	[145]
		NCT02807805	II	Metastatic Prostate/Recurrent Prostate Carcinoma, Stage IV Prostate Cancer	[146]
		NCT03123978	I	Metastatic Prostate/Recurrent Prostate Carcinoma, Stage IV Prostate Cancer	[147]
Frizzled 1, 2, 5, 7, 8 receptors	OMP-18R5 (Vanticumab)	NCT01957007	I	Solid Tumors	[148]
		NCT01973309	I	Metastatic Breast Cancer	[149]
		NCT01345201	I	Solid Tumors	[150]
		NCT02005315	I	Pancreatic/Stage IV Pancreatic Cancer	[151]
Frizzled 10 receptor	OTSA101	NCT04176016	I	Relapsed or Refractory Synovial Sarcoma	[152]
		NCT01469975	I	Sarcoma, Synovial	[153]
ROR1 receptor	UC-961 (Cirmtuzumab)	NCT02860676	I	Chronic Lymphocytic Leukemia	[154]
		NCT02222688	I	Chronic Lymphocytic Leukemia	[155]
		NCT02776917	I	Breast Neoplasms	[156]
		NCT03088878	I|II	B-cell Chronic Lymphocytic Leukemia, Small Lymphocytic Lymphoma, Mantle Cell Lymphoma	[157]
		NCT04501939	II	Chronic Lymphocytic Leukemia	[158]
β-catenin degradation	CWP232291 (CWP291)	NCT02426723	I	Multiple Myeloma	[159]
		NCT03055286	I|II	Acute Myeloid Leukemia	[160]
		NCT01398462	I	Acute Myeloid Leukemia, Chronic Myelomonocytic Leukemia, Myelodysplastic Syndrome, Myelofibrosis	[161]
CBP/β-catenin interaction	PRI-724	NCT02413853	II	Colorectal Adenocarcinoma, Stage IVA/Stage IVB Colorectal Cancer	[162]
		NCT01302405	I	Advanced Solid Tumors	[163]
		NCT01606579	I|II	Acute/Chronic Myeloid Leukemia	[164]
		NCT01764477	I	Advanced Pancreatic/Metastatic Pancreatic Cancer, Pancreatic Adenocarcinoma	[165]
CLK inhibition	SM08502	NCT03355066	I	Solid Tumor, Adult	[166]
